# Texture analysis of magnetic resonance image to differentiate benign from malignant myxoid soft tissue tumors: A retrospective comparative study

**DOI:** 10.1371/journal.pone.0267569

**Published:** 2022-05-19

**Authors:** Hyunsik Chang, Yusuhn Kang, Joong Mo Ahn, Eugene Lee, Joon Woo Lee, Heung Sik Kang

**Affiliations:** Department of Radiology, Seoul National University Bundang Hospital, Bundang-gu, Seongnam-si, Gyeonggi-do, Korea; Mie University Graduate School of Medicine, JAPAN

## Abstract

It is important to differentiate between benign and malignant myxoid tumors to establish the treatment plan, determine the optimal surgical extent, and plan postoperative surveillance, but differentiation may be complicated by imaging-feature overlap. Texture analysis is used for quantitative assessment of imaging characteristics based on mathematically calculated pixel heterogeneity and has been applied to the discrimination of benign from malignant soft tissue tumors (STTs). In this study, we aimed to assess the diagnostic value of the texture features of conventional magnetic resonance images for the differentiation of benign from malignant myxoid STTs. Magnetic resonance images of 39 patients with histologically confirmed myxoid STTs of the extremities were analyzed. Qualitative features were assessed and compared between the benign and malignant groups. Texture analysis was performed, and texture features were selected based on univariate analysis and Fisher’s coefficient. The diagnostic value of the texture features was assessed using receiver operating curve analysis. T1 heterogeneity showed a statistically significant difference between benign and malignant myxoid STTs, with substantial inter-reader reliability. The sensitivity, specificity, positive predictive value, negative predictive value, and accuracy of T1 heterogeneity were 55.6%, 83.3%, 88.2%, 45.5%, and 64.1%, respectively. Among the texture features, T2w-WavEnLL_s-3 showed good diagnostic performance, and T2w-WavEnLL_s-4 and GeoW4 showed fair diagnostic performance. The logistic regression model including T1 heterogeneity and T2_WavEnLL_s-4 showed good diagnostic performance. However, there was no statistically significant difference between the overall qualitative assessment by a radiologist and the predictor model. Geometry-based and wavelet-derived texture features from T2-weighted images were significantly different between benign and malignant myxoid STTs. However, the texture features had a limited additive value in differentiating benign from malignant myxoid STTs.

## Introduction

Myxoid soft tissue tumors (STTs) are a heterogeneous group of mesenchymal tumors characterized by an abundant production of extracellular myxoid stroma [1–4]. The myxoid stroma tends to trap water molecules, resulting in the high-water content of myxoid STTs. Myxoid tumors share a common magnetic resonance imaging (MRI) feature of low signal intensity on T1-weighted images (T1WIs) and high signal intensity on T2-weighted images (T2WIs) [5,6]. Despite the common histologic and imaging features, the biological behavior of these tumors varies widely, from benign to malignant.

The differentiation between benign and malignant myxoid tumors is crucial for establishing a proper treatment plan, determining the optimal surgical extent, and planning postoperative surveillance. Several imaging features, including tumor size, shape or margin, presence of necrosis, edema, and homogeneity of T1 signal intensity, are known to aid in the differentiation between benign and malignant STTs [7–10]. However, there is an overlap of imaging features, making the differentiation difficult. This is particularly relevant for myxoid STTs, due to the common imaging features resulting from the high-water content.

Several studies have attempted to identify the imaging features differentiating benign from malignant myxoid tumors. Harish et al. [11] suggested that the diagnosis of malignancy was favored when the lesion exhibited a larger average dimension and heterogeneity on T1WIs. Crombe et al. [12] reported that the differentiation between benign and malignant lesions could be reproducibly achieved using conventional MR features, including ill-defined tumor margins, intratumoral hemorrhage or fat, fibrosis, and presence of the tail sign. Other studies have emphasized the importance of certain imaging signs in the diagnosis of myxoid STTs: the “shiny cap” sign for intramuscular myxomas [13], the “target sign” in peripheral nerve sheath tumors [14], and the “tail sign” in myxofibrosarcomas and undifferentiated sarcomas [15,16]. However, most of the suggested findings are qualitative imaging features, which are limited by their subjectivity.

Texture analysis is a novel imaging tool for quantitative assessment of imaging characteristics based on mathematically calculated pixel heterogeneity. In recent decades, many studies have reported promising results of texture analysis in the differentiation and prognosis of tumors of the brain, lung, liver, and soft tissue. Several studies have evaluated the use of texture analysis for discriminating benign from malignant STTs [8,17,18] and cartilaginous bone tumors [19]. We hypothesized that texture analysis of conventional MR images of myxoid STTs may aid in the differentiation of benign from malignant myxoid STTs.

Thus, the purpose of this study was to assess the diagnostic value of texture features of conventional MR images for differentiating between benign and malignant myxoid STTs of the musculoskeletal system and to evaluate their additive value to the qualitative assessment of MR images.

## Materials and methods

The Institutional Review Board of Seoul National University Bundang Hospital (IRB No. B-1910-568-102) approved this study and the requirement for informed consent was also waived by the Institutional Review Board of Seoul National University Bundang Hospital. Data for the study was retrospectively obtained and anonymized, posing no more than minimal risk to the study subjects. All data were anonymized before analysis and all methods were performed in accordance with the relevant guidelines and regulations.

### Study population

We searched our electronic medical records to identify patients with histologically confirmed myxoid STTs of the extremities who had undergone preoperative MRI between January 2010 and December 2018. The following myxoid STTs were included in our search: myxoma, myxolipoma, acral fibromyxoma, fibromyxoid tumor, myxoid liposarcoma, fibromyxoid sarcoma, myxoinflammatory fibroblastic sarcoma, myxofibrosarcoma, myxosarcoma, and extraskeletal myxoid chondrosarcoma. This search revealed 81 patients.

A second search was performed to identify patients with tumors that were initially assessed as “myxoid STT” on MRI but were histologically confirmed otherwise (n = 16); this included schwannomas (n = 10), neurofibromas (n = 4), cellular angiofibroma (n = 1), and nodular fasciitis (n = 1).

Among the 97 patients, we excluded those who met any of the following exclusion criteria: 1) history of previous surgery (n = 6); 2) inadequate MR image quality due to low resolution or artifacts (n = 18); 3) MR images acquired with MR systems with a magnetic field strength of 1.5 T or lower (n = 15); and 4) insufficient pulse sequences for evaluation (n = 19).

### MRI protocol

All patients underwent MRI with 3-T MR scanners (Achieva or Ingenia 3T, Philips Healthcare, Best, The Netherlands) prior to tumor resection. Dedicated coils were used according to the tumor location. MR sequence parameters were as follows: T1W turbo spin-echo (TSE) axial and coronal scans—repetition time (TR), 400–750 ms; echo time (TE), 6–15 ms; flip angle (FA), 90°; echo train length (ETL), 4–8; T2W TSE axial, coronal, and sagittal scans—TR, 2,000–3,500 ms; TE, 35–100 ms; FA, 90°; ETL 12–18; TSE short tau inversion recovery axial scans (STIR)—TR, 2,200–3,500 ms; TE, 65–100 ms; FA, 90°; ETL 12–18; contrast-enhanced T1W fat-saturated axial, coronal, and sagittal scans—TR, 500–750 ms; TE, 6–15 ms; FA, 90°; ETL 4–8. The section thickness, intersection gap, and field of view were 2.5–6 mm, 0.25–1 mm, 100 × 100–690 × 430 mm, respectively.

### Qualitative assessment of MRI features

Two readers (a radiologist with 8 years of experience in musculoskeletal radiology, and a radiologist in musculoskeletal radiology fellowship training) independently evaluated the MR images using a picture archiving communication system (INFINITT; Infinitt Healthcare, Seoul, Korea). The readers were aware of the patients’ clinical information, including age, sex, and clinical history before surgery, but were blinded to the histological diagnosis.

The following tumor characteristics were assessed: 1) tumor size, measured as the longest diameter of the tumor in the axial, coronal, or sagittal plane (equal to or larger than 5 cm; less than 5 cm); 2) tumor margin (well defined; ill-defined or irregular); 3) depth, defined as the deepest portion of the tumor extent (subcutaneous; extending to or involving the deep peripheral fascia); 4) involved compartment (unicompartmental; multicompartmental); 5) homogeneity of signal intensity on T1WIs (homogeneous; heterogeneous); 6) homogeneity of signal intensity on T2WIs (homogeneous; heterogeneous); 7) intratumoral hemorrhage (absent; present); 8) intratumoral septation (absent; present); 9) intratumoral necrosis (absent; present); 10) peritumoral edema (absent; present); and 11) fascial tail sign (absent; present).

In addition, each reader was asked to determine whether the STTs were benign or malignant, considering the above-mentioned MRI characteristics (overall assessment).

### Texture analysis

**Fig 1:**
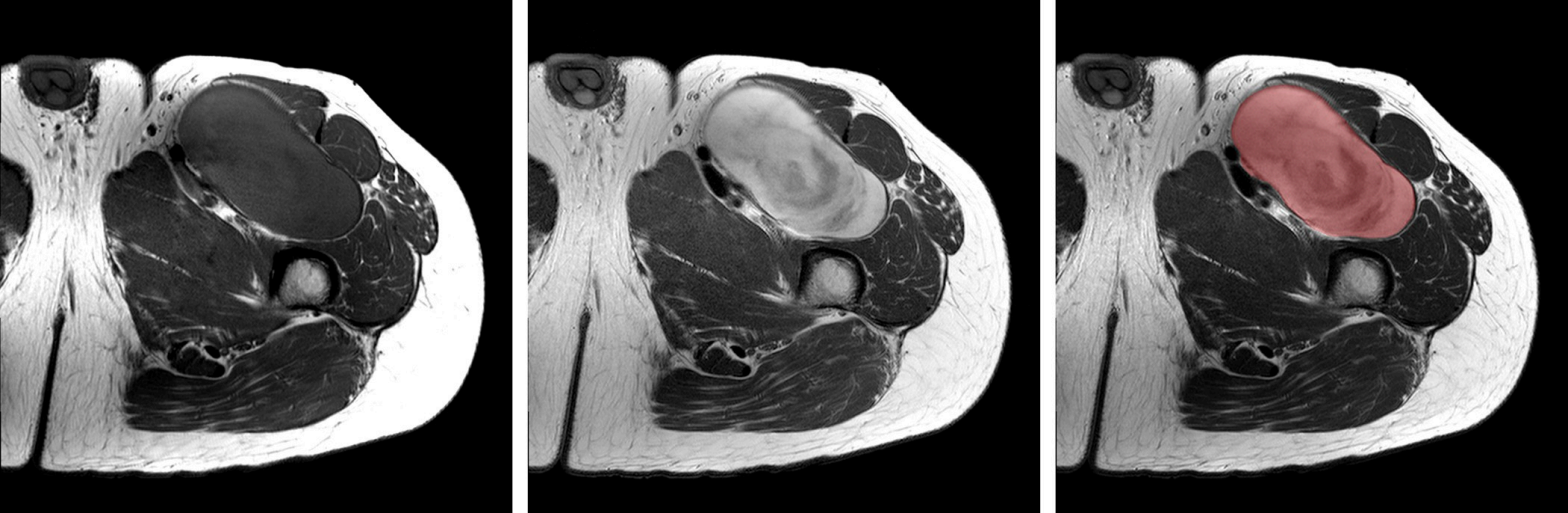
A 36-year-old man with a thigh mass. (a) Axial T1- and (b) T2WIs showing an intermuscular mass with well-defined margins. The mass appears homogeneous and isointense to slightly hyperintense to adjacent muscles on the T1WI and mildly heterogeneous and hyperintense on the T2WI. (c) A region of interest selected for analysis on the axial T2WI. (d) Texture features extracted from the selected region of interest. This mass was histologically confirmed as a neurofibroma. T1WI: T1-weighted image.

**Fig 2:**
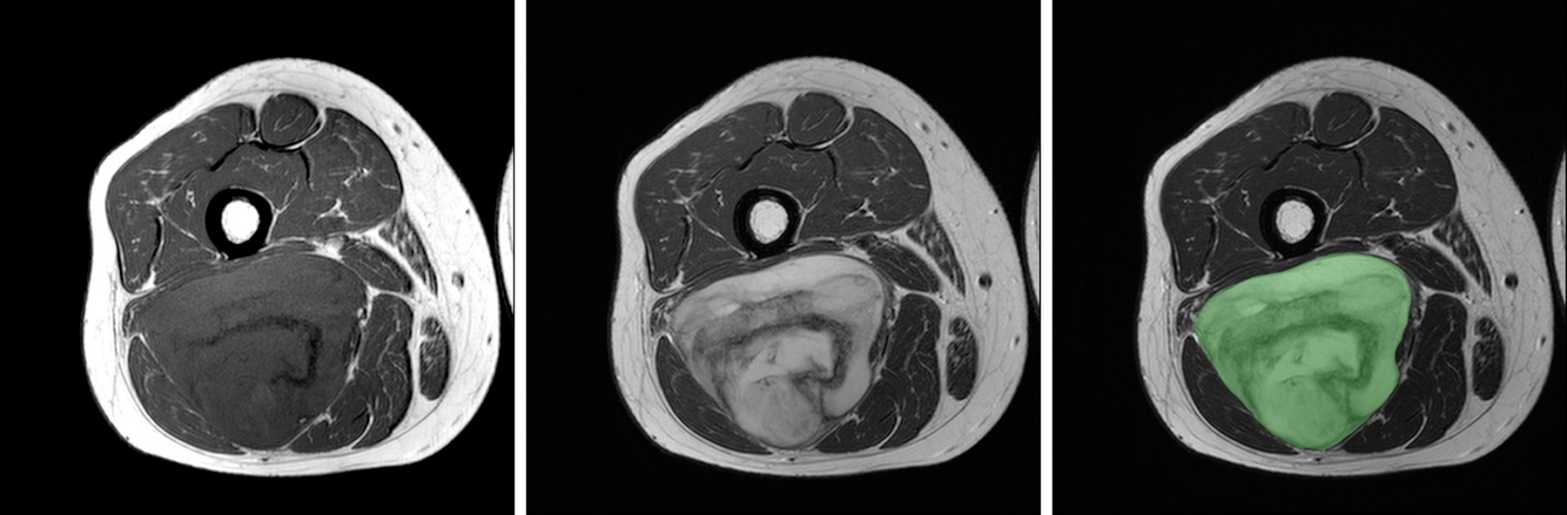
A 34-year-old woman with a thigh mass. (a) Axial T1- and (b) T2WIs showing an intermuscular mass in the posterior thigh compartment. The mass appears heterogeneous on the T1WI with subtle hyperintensity, possibly resulting from intratumoral hemorrhage. The curved linear dark signal intensity (arrowheads) resulted from metaplastic bone formation within the mass. (b) On the axial T2WI, the lesion appears heterogeneous and hyperintense. Curved linear dark signal intensity (arrowheads) is also noted. (c) A region of interest selected for analysis on the axial T2WI. (d) Texture features extracted from the selected region of interest. This mass was histologically confirmed as a myxoid liposarcoma. T1WI: T1-weighted image.

For each lesion, the radiologist in fellowship training selected a single axial image section that best represented the characteristics of the tumor both on T1WIs and T2Wis (Figs 1 and 2), and a single region of interest (ROI) was drawn manually along the tumor border (Figs 1C and 2C). In case of suboptimal image quality of the axial images resulting from motion artifacts from pulsating vessels, coronal or sagittal images were used for ROI selection. The second, experienced radiologist reviewed and confirmed the selected free-hand ROIs.

For texture analysis of myxoid STTs, we used the software package MaZda 4.6 (Institute of Electronics, Technical University of Lodz, Poland; available at http://www.eletel.p.lodz.pl/mazda/), which can calculate more than 300 texture features [20–22]. The signal intensity of each ROI was normalized prior to the computation of textural features, using the limitation of dynamics to μ ± 3 σ (μ, gray-level mean; σ, gray-level standard deviation) to reduce dependency of higher order features on first-order gray-level distribution.

The following geometry and texture features were computed for each ROI: geometry features (horizontal and vertical coordinate of gravity center, maximal diameter, perimeter, etc.), gray-level histogram features (mean, variance, skewness, kurtosis, percentiles 1%, 10%, 50%, 90%, and 99%); absolute gradient features (mean, variance, skewness, kurtosis, and percentage of pixels with nonzero); the co-occurrence matrix (angular second moment, contrast, correlation, sum of squares, inverse difference moment, sum average, sum variance, sum entropy, entropy, difference variance, and difference entropy), which was computed for five between-pixels distances (1–5) and for four directions (horizontal, vertical, 45 degrees, and 135 degrees); the run-length matrix (run-length nonuniformity, gray-level nonuniformity, long-run emphasis, short-run emphasis, and fraction of image in runs), which was computed for four directions (horizontal, vertical, 45 degrees, and 135 degrees); the autoregressive model (Theta: model parameter vector, four features; Sigma: standard deviation of the driving noise); the wavelet-derived features (wavelet energy), which was computed at five scales within four frequency bands {low-low(LL), low-high(LH), high-low(HL), and high-high(HH)}.

The geometry and texture features that were among the top 10 features selected based on Fisher’s coefficient and showed a significant between-group difference in the univariate analysis were selected for further analysis.

### Statistical analysis

Categorical variables were compared using either the chi-square test or Fisher’s exact test. Continuous variables were tested for normality using the Shapiro–Wilk test, and the groups were compared using either the independent Student’s t-test or the rank sum test, as appropriate. For texture features showing a significant difference between the benign and malignant groups, receiver operating curve (ROC) analysis was performed to determine their diagnostic value.

To create a model for prediction of malignancy, logistic regression analysis was performed including MRI findings and texture features. ROC comparison was performed by radiologists to compare the diagnostic performance of the logistic regression model with that of the overall qualitative assessment using the method suggested by DeLong et al. [23]. The discriminatory power was classified based on the area under the curve (AUC) as follows: 0.9–1, excellent; 0.8–0.9, good; 0.7–0.8, fair; 0.6–0.7, poor; and 0.5–0.6, failure.

Inter-reader agreement was assessed using weighted kappa (κ) statistics. According to Landis and Koch [24], the κ values were categorized as follows: 0–0.2, slight; 0.21–0.4, fair; 0.41–0.60, moderate; 0.61–0.80, substantial; and 0.81–1.0, excellent agreement.

Statistical analyses were performed using IBM SPSS Statistics v.21.0 (IBM Corp., Armonk, NY, USA) and STATA (v.14.0; Stata, College Station, TX, USA). A *p*-value of <0.05 was considered statistically significant.

## Results

A total of 39 patients (21 men and 18 women) with a mean age of 54.8±13.8 years (range, 19–87 years) were included in this study. The demographic features, histologic diagnoses, and lesion locations are listed in Table 1.

**Table 1:** Demographic features.

	Benign (n = 12)	Malignant (n = 27)
**Age (years)**	52.3 ± 11.7	55.9 ± 14.6
**Gender**		
Male	4 (33.3%)	17 (67.0%)
Female	8 (66.7%)	10 (37.0%)
**Lesion Location**		
Lower limb	4 (33.3%)	16 (59.3%)
Pelvic girdle	2 (16.7%)	5 (18.5%)
Upper limb	4 (33.3%)	5 (18.5%)
Shoulder girdle	2 (16.7%)	1 (3.7%)
**Histologic type**	Intramuscular myxoma (n = 8)	Myxoid liposarcoma (n = 11)
	BPNST (n = 3)	Myxofibrosarcoma (n = 10)
	Neurofibroma (n = 1)	Low-grade fibromyxoid sarcoma (n = 3)
		Myxoinflammatory fibroblastic sarcoma (n = 1)
		Low-grade myofibroblastic sarcoma (n = 1)
		Extraskeletal myxoid chondrosarcoma (n = 1)

### Qualitative assessment of MRI features

The qualitative MRI features for discriminating benign from malignant myxoid STTs and their inter-reader reliability are presented in Table 2. Among them, only T1 heterogeneity showed a statistically significant difference between benign and malignant myxoid STTs (*p* = 0.024) in the univariate analysis, and the inter-reader reliability was substantial (κ = 0.679). The sensitivity, specificity, positive predictive value, negative predictive value, and accuracy for discriminating benign from malignant myxoid STTs were 55.6%, 83.3%, 88.2%, 45.5%, and 64.1%, respectively, for T1 heterogeneity and 88.9%, 50.0%, 80.0%, 66.6%, and 76.9%, respectively, for the overall assessment.

**Table 2:** Univariate analysis of conventional MRI features for differentiating between benign and malignant myxoid soft tissue tumors.

MRI feature	Benign (n = 12)	Malignant (n = 27)	P value	Interobserver agreement (κ)
**Size**				
Maximal dimension (cm)	6.2 ± 3.9	7.6 ± 3.4	0.267 ^a^	
Size > 5cm	6 (50.0%)	21 (77.8%)	0.133 ^b^	0.941
**Location**			0.285 ^b^	0.713
Subcutaneous	1 (8.3%)	9 (33.3%)		
Intramuscular	8 (66.7%)	12 (44.4%)		
Intermuscular	3 (25.0%)	6 (22.2%)		
**Multicompartmental**	1 (8.3%)	6 (22.2%)	0.403 ^b^	0.687
**Margin**			0.645 ^b^	0.414
Well-defined margin	11 (91.7%)	22(81.5%)		
Irregular, infiltrative margin	1 (8.3%)	5 (18.5%)		
**Intratumoral features**				
T1 heterogeneity	2 (16.7%)	15 (55.6%)	0.037 ^b^	0.679
T2 heterogeneity	9 (75.0%)	24 (88.9%)	0.348 ^b^	0.363
Hemorrhage	0 (0%)	5 (18.5%)	0.299 ^b^	0.541
Necrosis	3 (25.0%)	11 (40.7%)	0.477 ^b^	0.591
**Peritumoral features**				
Peritumoral edema	7 (58.3%)	10 (37.0%)	0.299 ^b^	0.492
Fascial tail	2 (16.7%)	6 (22.2%)	1.000 ^b^	0.424

Note–Data are presented as the number of cases with percentages in parenthesis. MRI, magnetic resonance imaging.

^a^ t-test

^b^ Fisher’s exact test; significance level = 0.05.

### Texture analysis

The selected geometry and texture features of T1- and T2WIs are listed in Table 3, along with their diagnostic performance. Texture features were selected according to the results of the univariate analysis and their ranking based on Fisher’s coefficient. The selected features included contour-skeleton-derived features (GeoS2), perimeter-derived features (GeoW4, GeoW5b), and wavelet energy functions derived from T2WIs (T2-WavEnLL_s-3, T2-WavEnLL_s-4).

**Table 3:** Top 10 texture features for discrimination between benign and malignant myxoid soft tissue tumors on T1- and T2-weighted images based on Fischer’s coefficients and univariate analysis.

T1-weighted image		T2-weighted image	
Fisher coefficient	Univariate analysis	Fisher coefficient	Univariate analysis
GeoW5b (F = 0.97)	WavEnHL_s-5 (p = 0.003)	GeoS2 (F = 1.40)	GeoS2 (p = 0.004)
WavEnLH_s-4 (F = 0.63)	GeoW5b (p = 0.009)	GeoW5b (F = 1.01)	GeoW5b (p = 0.008)
GeoS2 (F = 0.60)	GeoW1 (p = 0.011)	WavEnLL_s-4 (F = 0.89)	WavEnLL_s-4 (p = 0.012)
GeoW2 (F = 0.39)	GeoW4 (p = 0.026)	WavEnLL_s-3 (F = 0.87)	WavEnLL_s-3 (p = 0.012)
GeoRc (F = 0.37)	GeoS2 (p = 0.032)	WavEnLL_s-2 (F = 0.59)	WavEnLL_s-5 (p = 0.014)
GeoRm (F = 0.34)	WavEnLH_s-5 (p = 0.064)	WavEnLH_s-2 (F = 0.48)	WavEnLL_s-2 (p = 0.036)
WavEnLH_s-3 (F = 0.34)	GeoW3 (p = 0.088)	GeoXo (F = 0.45)	GeoW4 (p = 0.041)
GeoW4 (F = 0.34)	GeoRs (p = 0.088)	WavEnLL_s-1 (F = 0.43)	GeoNi (p = 0.068)
GeoRs (F = 0.33)	GeoRc (p = 0.088)	GeoLsz (F = 0.38)	GeoNx (p = 0.068)
GeoW3 (F = 0.33)	GeoRm (p = 0.088)	GeoW4 (F = 0.38)	GeoXo (p = 0.069)

According to the AUC values, T2w-WavEnLL_s-3 (AUC, 0.890) showed good diagnostic performance, and T2w-WavEnLL_s-4 (AUC, 0.773) and GeoW4 (AUC, 0.737) showed fair diagnostic performance. Conversely, GeoS2 (AUC, 0.553) and GeoW5b (AUC, 0.587) showed diagnostic failure.

### Combined conventional and logistic regression model

The logistic regression model was constructed by including the selected morphologic and texture analysis features with the best diagnostic performance for differentiating benign from malignant myxoid STTs. Among all, the model including T1 heterogeneity and T2_WavEnLL_s-4 showed the highest AUC value and good diagnostic performance (AUC, 0.833; 95% confidence interval, 0.688–0.978; Table 4). However, there was no statistical difference in the AUC values between the overall assessment by a radiologist based on qualitative MR features and the combined model including T1 heterogeneity and T2_WavEnLL_s-4 (*p* = 0.085).

**Table 4:** Diagnostic performance of individual texture parameters for differentiating between benign and malignant myxoid soft tissue tumors.

Parameters	AUC (95% CI)	Cutoff	Sens (%)	Spec (%)	Correctly classified
**GeoS2**	0.680 (0.510–0.850)	≤ 0.5	77.8	58.3	71.8
**GeoW4**	0.725 (0.555–0.895)	≥ 2.70	85.2	58.3	76.9
**GeoW5b**	0.587 (0.390–0.784)	≤ 0.14	51.9	91.7	64.1
**T2-WavEnLL_s-4**	0.735 (0.561–0.908)	≥ 13799	74.1	66.7	71.8
**T2-WavEnLL_s-3**	0.735 (0.564–0.905)	≥ 15157	81.5	58.3	74.4
**Overall assessment based on qualitative MR features**	0.694 (0.535–0.854)	88.9	50.0	76.9	74.4
**Combined model with conventional and texture features**	0.833 (0.688–0.978)	77.8	83.3	79.5	

Note–The combined model with conventional and texture features included T1 heterogeneity and T2-WavEnLL_s-4 as predictors. AUC, area under the curve; CI, confidence interval; Sens, sensitivity; Spec, specificity.

## Discussion

Differentiation between benign and malignant myxoid STTs is often challenging because of their common imaging features resulting from the myxoid stroma. In this study, we focused on myxoid STTs and found that among various texture features derived from conventional T1- and T2WIs, geometry-based and wavelet-derived features showed a significant difference between benign and malignant myxoid STTs. However, they had no significant additive value in differentiating benign from malignant myxoid STTs relative to the overall qualitative assessment by a radiologist.

Numerous studies have addressed the differentiation between benign and malignant STTs [7,9,17,25–27]. Most studies have emphasized the importance of lesion size, margin irregularity, and heterogeneity of lesion signal intensity on either T1- or T2WIs, and others have attempted a more objective approach with texture analysis [17,26]. However, few studies have focused on the differentiation of myxoid tumors [11,12,18,28]. A recent study by Martin-Carreras et al [29] have shown that radiomic features from MRI are helpful in differentiating myxomas from myxofibrosarcomas, along with the T1-weighted signal intensity and volume of the lesion.

In our study, heterogeneity of T1 signal intensity was the sole qualitative imaging feature showing a significant difference between benign and malignant myxoid STTs. This finding coincides with that of Harish et al. [11], who found that heterogeneity of the lesion on T1WIs was an imaging feature favoring the diagnosis of malignancy in STTs with “cyst-like” appearance. However, in that study, a larger average dimension of the mass was statistically the most significant predictor of malignancy, which was not the case in our study. This discrepancy may have been caused by the difference in the inclusion criteria. Namely, Harish et al. included lesions based on their signal intensity, and the benign group included ganglion and bursa lesions, as well as myxomas and schwannomas. In our study, we did not include overtly benign lesions with typical imaging features, such as ganglion and bursa lesions or schwannomas; we only included those benign lesions that may pose a diagnostic challenge. As a result, there was no statistically significant difference in the lesion size between the benign and malignant groups.

In the present study, imaging features such as lesion margin, intratumoral hemorrhage, necrosis, or peritumoral features, including peritumor edema and fascial tail sign, did not show a significant difference between benign and malignant lesions. This is conflicting with the findings of Crombe et al. [12], who showed that ill-defined margins, intratumoral fat, a hemorrhagic component, fibrosis, and the “tail sign” were associated with malignancy. The majority of both benign and malignant lesions in our study showed a well-defined border, which was also found in the study by Harish et al [11]. This shows that the assessment of qualitative imaging features may be subjective and limited in terms of reproducibility, emphasizing the need for an objective, quantitative assessment of imaging findings. Hence, we applied texture analysis for a more objective assessment of the intratumoral signal intensities on T1- and T2WIs.

Several texture features derived from geometry and wavelet energy of T1WIs and T2WIs showed a significant difference between benign and malignant myxoid tumors. However, these differences were small and showed low Fisher’s coefficients, indicating a low discriminating value. These results are similar to the findings reported in previous studies. Mayerhoefer et al. [30] investigated the differentiation of benign from malignant STTs on MRI by means of texture analysis. They included a heterogeneous group of benign non-neoplastic, benign neoplastic, and malignant lesions, and found that only two texture features derived from the gray-level histogram of short-tau inversion recovery sequences were able to discriminate between benign and malignant lesions. Contrarily, Juntu et al. [17] applied machine learning to the texture analysis features of T1WIs and concluded that it is a potentially valuable tool for the differentiation between malignant and benign STTs. In their study, machine learning classifiers based on texture features showed an accuracy of up to 93%, which was higher than the radiologist classification accuracy of 90%. However, most of the benign tumors included in their study seldom pose a diagnostic problem: lipomas, schwannomas, and cavernous hemangiomas. In our study, the overall assessment by a radiologist based on qualitative imaging features showed a diagnostic accuracy of 69.4%. The relatively low diagnostic accuracy probably resulted from the inclusion of only diagnostically challenging cases in the benign lesion group. Furthermore, both the individual texture features and the combined logistic model failed to show a significant increase in the diagnostic performance compared with the overall assessment by the radiologist. Texture features derived from conventional MR images seem to have a limited additive value in the differentiation of benign from malignant myxoid STTs in cases that are diagnostically challenging to radiologists.

There are some limitations in our study. First, it was performed retrospectively and only included a small number of histologically confirmed cases. Thus, future prospective studies with a larger sample size are warranted. Second, because of the retrospective design, the MRI protocol varied. The non-standardized imaging parameters (TR, TE, and pixel size) may have affected the texture features. Further studies with a more standardized imaging protocol may be needed. Third, contrast-enhanced images were not included. We excluded contrast-enhanced images from our analysis because the amount of administered contrast medium and the time interval between contrast medium injection and image acquisition varied from case to case, which would have affected the texture features. Finally, we used a two-dimensional approach for texture analysis. As complete three-dimensional volume data were not included, the results could vary depending on the image slice selected for analysis.

## Conclusions

In conclusion, geometry-based and wavelet-derived texture features from T2WIs showed a significant difference between benign and malignant myxoid STTs. However, the texture features of T1- and T2WIs had a limited additive value in differentiating benign from malignant myxoid STTs relative to the overall qualitative image assessment by radiologists.

## Supporting information

S1 Table Texture parameter analysis. (XLSX)

## References

[1] Allen PW. Myxoid tumors of soft tissues. Pathol Annu. 1980;15(Pt 1):133–92. 7443305

[2] Graadt van Roggen JF, Hogendoorn PC, Fletcher CD. Myxoid tumours of soft tissue. Histopathology. 1999;35(4):291–312. doi: 10.1046/j.1365-2559.1999.00835.x 10564384

[3] Mackenzie DH. The myxoid tumors of somatic soft tissues. Am J Surg Pathol. 1981;5(5):443–58. doi: 10.1097/00000478-198107000-00004 6269445

[4] Tarabishy Y, Pittman ME. Soft Tissue Tumors With Myxoid Stroma: A Review of Distinguishing Clinical and Pathologic Features. Ajsp-Rev Rep. 2017;22(2):94–101.

[5] Petscavage-Thomas JM, Walker EA, Logie CI, Clarke LE, Duryea DM, Murphey MD. Soft-tissue myxomatous lesions: review of salient imaging features with pathologic comparison. Radiographics. 2014;34(4):964–80. doi: 10.1148/rg.344130110 25019435

[6] Walker EA, Fenton ME, Salesky JS, Murphey MD. Magnetic resonance imaging of benign soft tissue neoplasms in adults. Radiol Clin North Am. 2011;49(6):1197–217, vi. doi: 10.1016/j.rcl.2011.07.007 22024295

[7] Berquist TH, Ehman RL, King BF, Hodgman CG, Ilstrup DM. Value of MR imaging in differentiating benign from malignant soft-tissue masses: study of 95 lesions. AJR Am J Roentgenol. 1990;155(6):1251–5. doi: 10.2214/ajr.155.6.2122675 2122675

[8] Chen CK, Wu HT, Chiou HJ, Wei CJ, Yen CH, Chang CY, et al. Differentiating benign and malignant soft tissue masses by magnetic resonance imaging: role of tissue component analysis. J Chin Med Assoc. 2009;72(4):194–201. doi: 10.1016/S1726-4901(09)70053-X 19372075

[9] Crim JR, Seeger LL, Yao L, Chandnani V, Eckardt JJ. Diagnosis of soft-tissue masses with MR imaging: can benign masses be differentiated from malignant ones? Radiology. 1992;185(2):581–6. doi: 10.1148/radiology.185.2.1410377 1410377

[10] Kransdorf MJ, Jelinek JS, Moser RP Jr., Utz JA, Brower AC, Hudson TM, et al. Soft-tissue masses: diagnosis using MR imaging. AJR Am J Roentgenol. 1989;153(3):541–7. doi: 10.2214/ajr.153.3.541 2763953

[11] Harish S, Lee JC, Ahmad M, Saifuddin A. Soft tissue masses with "cyst-like" appearance on MR imaging: Distinction of benign and malignant lesions. Eur Radiol. 2006;16(12):2652–60. doi: 10.1007/s00330-006-0267-5 16670867

[12] Crombe A, Alberti N, Stoeckle E, Brouste V, Buy X, Coindre JM, et al. Soft tissue masses with myxoid stroma: Can conventional magnetic resonance imaging differentiate benign from malignant tumors? Eur J Radiol. 2016;85(10):1875–82. doi: 10.1016/j.ejrad.2016.08.015 27666630

[13] Bancroft LW, Kransdorf MJ, Menke DM, O’Connor MI, Foster WC. Intramuscular myxoma: characteristic MR imaging features. AJR Am J Roentgenol. 2002;178(5):1255–9. doi: 10.2214/ajr.178.5.1781255 11959742

[14] Banks KP. The target sign: extremity. Radiology. 2005;234(3):899–900. doi: 10.1148/radiol.2343030946 15734940

[15] Kaya M, Wada T, Nagoya S, Sasaki M, Matsumura T, Yamaguchi T, et al. MRI and histological evaluation of the infiltrative growth pattern of myxofibrosarcoma. Skeletal Radiol. 2008;37(12):1085–90. doi: 10.1007/s00256-008-0542-4 18629459

[16] Yoo HJ, Hong SH, Kang Y, Choi JY, Moon KC, Kim HS, et al. MR imaging of myxofibrosarcoma and undifferentiated sarcoma with emphasis on tail sign; diagnostic and prognostic value. Eur Radiol. 2014;24(8):1749–57. doi: 10.1007/s00330-014-3181-2 24889995

[17] Juntu J, Sijbers J, De Backer S, Rajan J, Van Dyck D. Machine learning study of several classifiers trained with texture analysis features to differentiate benign from malignant soft-tissue tumors in T1-MRI images. J Magn Reson Imaging. 2010;31(3):680–9. doi: 10.1002/jmri.22095 20187212

[18] Kim HS, Kim JH, Yoon YC, Choe BK. Tumor spatial heterogeneity in myxoid-containing soft tissue using texture analysis of diffusion-weighted MRI. PLoS One. 2017;12(7):e0181339. doi: 10.1371/journal.pone.0181339 28708850 PMC5510859

[19] Fritz B, Muller DA, Sutter R, Wurnig MC, Wagner MW, Pfirrmann CWA, et al. Magnetic Resonance Imaging-Based Grading of Cartilaginous Bone Tumors: Added Value of Quantitative Texture Analysis. Invest Radiol. 2018;53(11):663–72. doi: 10.1097/RLI.0000000000000486 29863601

[20] Zhang Z, Song C, Zhang Y, Wen B, Zhu J, Cheng J. Apparent diffusion coefficient (ADC) histogram analysis: differentiation of benign from malignant parotid gland tumors using readout-segmented diffusion-weighted imaging. Dento maxillo facial radiology. 2019:20190100. doi: 10.1259/dmfr.20190100 31265331 PMC6775791

[21] Strzelecki M, Szczypinski P, Materka A, Klepaczko A. A software tool for automatic classification and segmentation of 2D/3D medical images. Nuclear Instruments and Methods in Physics Research Section A: Accelerators, Spectrometers, Detectors and Associated Equipment. 2013;702:137–40.

[22] Szczypinski PM, Strzelecki M, Materka A, Klepaczko A. MaZda—a software package for image texture analysis. Comput Methods Programs Biomed. 2009;94(1):66–76. doi: 10.1016/j.cmpb.2008.08.005 18922598

[23] DeLong ER, DeLong DM, Clarke-Pearson DL. Comparing the areas under two or more correlated receiver operating characteristic curves: a nonparametric approach. Biometrics. 1988;44(3):837–45. 3203132

[24] Landis JR, Koch GG. The measurement of observer agreement for categorical data. Biometrics. 1977;33(1):159–74. 843571

[25] Soler R, Castro JM, Rodríguez E. Value of MR findings in predicting the nature of the soft tissue lesions: benign, malignant or undetermined lesion? Computerized medical imaging and graphics: the official journal of the Computerized Medical Imaging Society. 1996;20(3):163–9. doi: 10.1016/0895-6111(96)00049-3 8930469

[26] Mayerhoefer ME, Breitenseher M, Amann G, Dominkus M. Are signal intensity and homogeneity useful parameters for distinguishing between benign and malignant soft tissue masses on MR images? Objective evaluation by means of texture analysis. Magn Reson Imaging. 2008;26(9):1316–22. doi: 10.1016/j.mri.2008.02.013 18448302

[27] Calleja M, Dimigen M, Saifuddin A. MRI of superficial soft tissue masses: analysis of features useful in distinguishing between benign and malignant lesions. Skeletal Radiol. 2012;41(12):1517–24. doi: 10.1007/s00256-012-1385-6 22491777

[28] Crombe A, Loarer FL, Alberti N, Buy X, Stoeckle E, Cousin S, et al. Homogeneous myxoid liposarcomas mimicking cysts on MRI: A challenging diagnosis. Eur J Radiol. 2018;102:41–8. doi: 10.1016/j.ejrad.2018.03.003 29685543

[29] Martin-Carreras T, Li H, Cooper K, Fan Y, Sebro R. Radiomic features from MRI distinguish myxomas from myxofibrosarcomas. BMC Med Imaging. 2019;19(1):67. doi: 10.1186/s12880-019-0366-9 31416421 PMC6694512

[30] Abaricia S, Hirbe AC. Diagnosis and Treatment of Myxoid Liposarcomas: Histology Matters. Curr Treat Options Oncol. 2018;19(12):64. doi: 10.1007/s11864-018-0590-5 30362022

